# A Rare Extramedullary Presentation of Acute Myeloid Leukemia: Myeloid Sarcoma Presenting as a Hard Scrotal Swelling

**DOI:** 10.7759/cureus.105945

**Published:** 2026-03-26

**Authors:** Ravindran Chirukandath, Sumin V Sulaiman, Akhtar Zamil Sirajudeen, Rajesh M Subramanian, Ayana Anil, Anagha Sukumar

**Affiliations:** 1 General Surgery, Government Medical College and Hospital, Thrissur, Thrissur, IND

**Keywords:** acute myeloid leukemia, extramedullary acute myeloid leukemia, hematologic neoplasms, myeloid neoplasm, scrotal swelling, testicular myeloid sarcoma

## Abstract

Myeloid sarcoma is an uncommon extramedullary tumor composed of immature myeloid precursor cells that occurs in association with acute myeloid leukemia (AML) and other myeloid neoplasms. These tumors may arise before, concurrently with, or after the diagnosis of systemic leukemia and can involve numerous anatomical locations, including the skin, lymph nodes, gastrointestinal tract, and soft tissues. Involvement of the scrotum or testis is particularly rare and often presents diagnostic challenges due to its ability to mimic inflammatory or primary neoplastic conditions.

We describe a case of a 68-year-old male who presented with persistent scrotal swelling initially treated as a scrotal abscess. Despite repeated incision and drainage procedures, the lesion persisted and was later accompanied by multiple cutaneous nodules involving the torso and lower extremities. Imaging demonstrated a heterogeneously enhancing lesion involving the scrotal wall with extension into the inguinal canal and associated lymphadenopathy. Histopathological examination with immunohistochemistry revealed tumor cells positive for myeloperoxidase and CD34 with a high Ki-67 proliferation index, confirming the diagnosis of multifocal myeloid sarcoma.

The patient received induction chemotherapy with cytarabine and daunorubicin along with localized radiotherapy. However, the clinical course was complicated by febrile neutropenia and multiorgan failure, resulting in death four weeks after initiation of therapy.

This case highlights the importance of considering hematologic malignancies in patients presenting with atypical or treatment-refractory scrotal swellings. Early biopsy and multidisciplinary evaluation are essential for timely diagnosis and appropriate management.

## Introduction

Myeloid sarcoma, historically referred to as granulocytic sarcoma or chloroma, is a solid tumor composed of immature myeloid precursor cells occurring outside the bone marrow. It is most commonly associated with acute myeloid leukemia (AML), although it may also arise in patients with myelodysplastic syndromes or myeloproliferative neoplasms. In certain cases, myeloid sarcoma may precede the development of systemic leukemia and serve as the initial manifestation of the disease [1,2].

Extramedullary involvement occurs in approximately 2-8% of patients with AML and may involve diverse anatomical locations such as the skin, lymph nodes, orbit, bone, central nervous system, and gastrointestinal tract. Among these sites, involvement of the scrotum or testis is extremely uncommon [3]. Most available information regarding testicular or scrotal myeloid sarcoma originates from isolated case reports or small case series, highlighting the rarity of this clinical presentation [3-5].

Scrotal swellings are frequently caused by benign or infectious conditions, including hydrocele, epididymitis, orchitis, scrotal abscess, or primary testicular tumors. Because these diagnoses are far more common, rare hematologic malignancies involving the scrotal region may initially be overlooked, leading to delays in definitive diagnosis and treatment [4,6].

In this report, we present a rare case of multifocal myeloid sarcoma presenting as persistent scrotal swelling initially managed as a scrotal abscess. The case emphasizes the diagnostic challenges associated with this unusual presentation and underscores the importance of early tissue biopsy in atypical scrotal lesions.

## Case presentation

A 68-year-old male with a known history of type 2 diabetes mellitus, controlled with insulin, presented with progressive scrotal swelling of approximately two months’ duration. Prior to the presentation at our institution, the patient had been treated at several local healthcare facilities, where the swelling was diagnosed as a scrotal abscess. He underwent multiple incisions and drainage procedures; however, the swelling persisted, and purulent discharge continued from the surgical sites.

**Figure 1:**
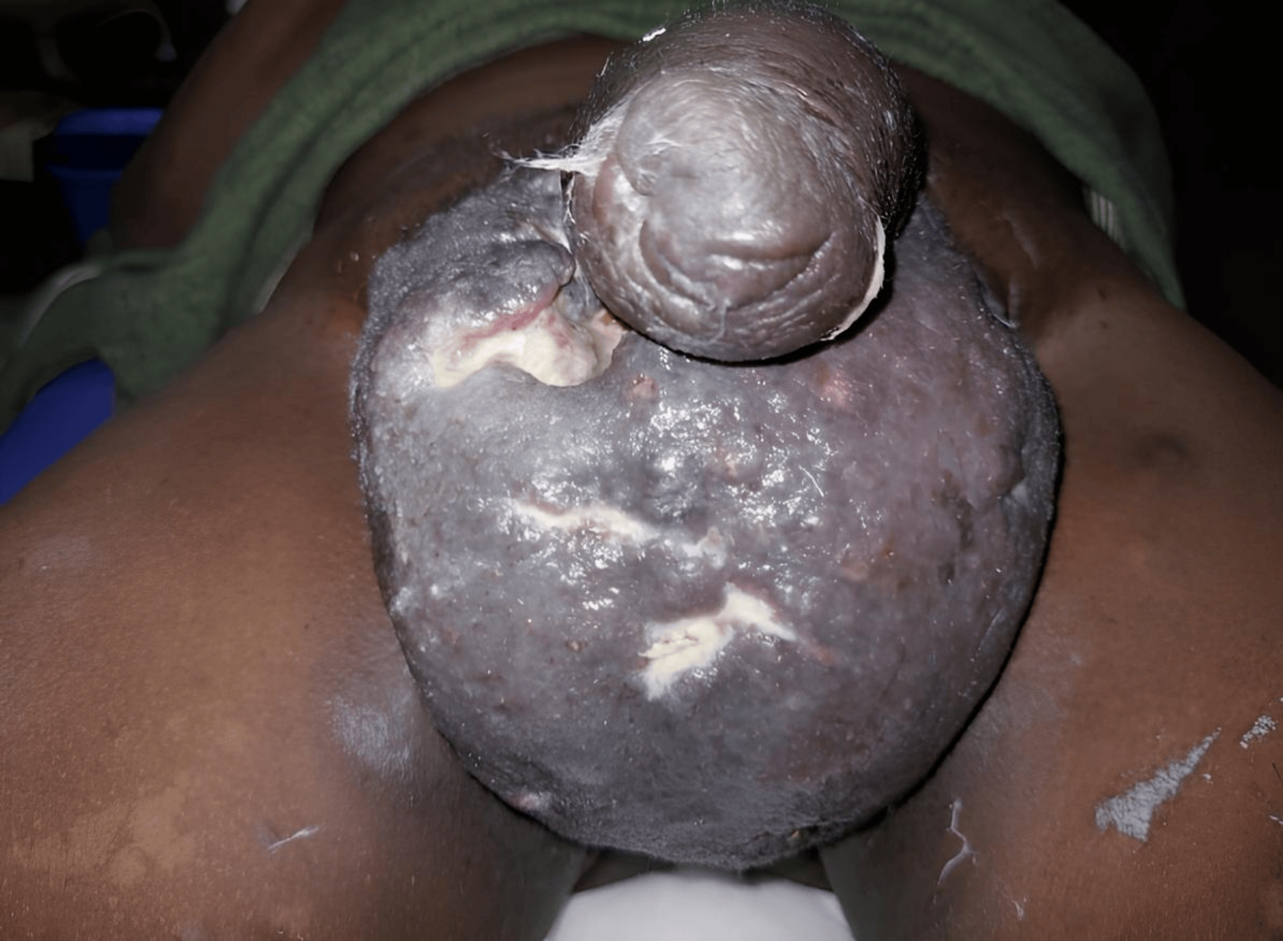
Scrotal swelling with marked induration.

On physical examination, the scrotum was diffusely enlarged, measuring approximately 10 × 10 cm. Figure 1 shows the scrotal wall as markedly indurated with a firm, woody consistency. Due to the pronounced thickening of the scrotal tissues, the testes could not be distinctly palpated. Local tenderness was present.

**Figure 2:**
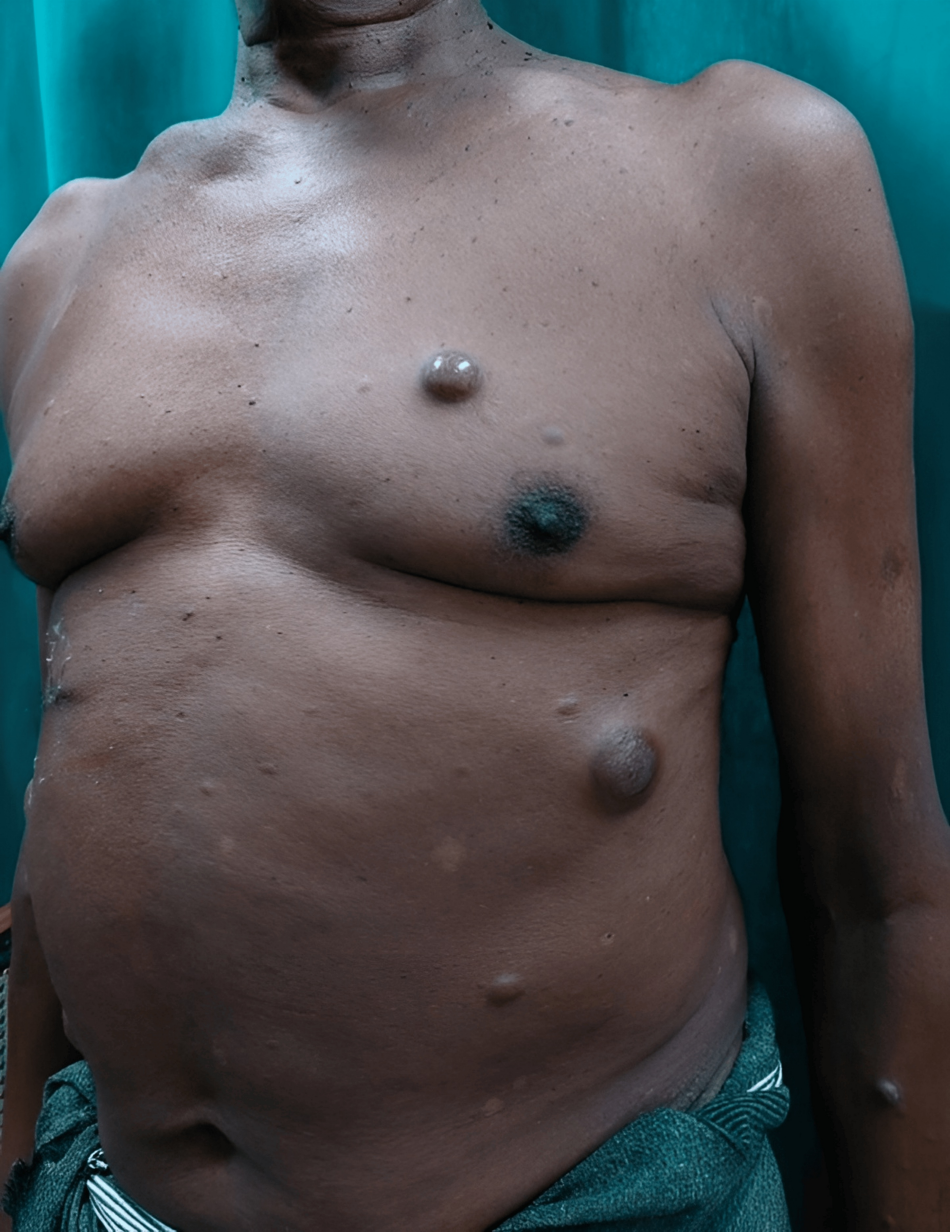
Tender cutaneous nodules over the torso.

Two days after admission, the patient developed multiple tender cutaneous nodules over the torso and lower extremities, as seen in Figure 2. These nodules measured approximately 2 × 2 cm and were firm in consistency.

Laboratory investigations revealed a hemoglobin level of 12.5 g/dL. CRP was elevated with a negative blood culture. A wound swab taken from the site was also found to be negative. Other routine hematological parameters were within normal limits at the time of initial evaluation.

Ultrasonography of the scrotum demonstrated diffuse thickening of the scrotal wall and subcutaneous tissues with increased echogenicity. Both testes appeared structurally normal.

**Figure 3:**
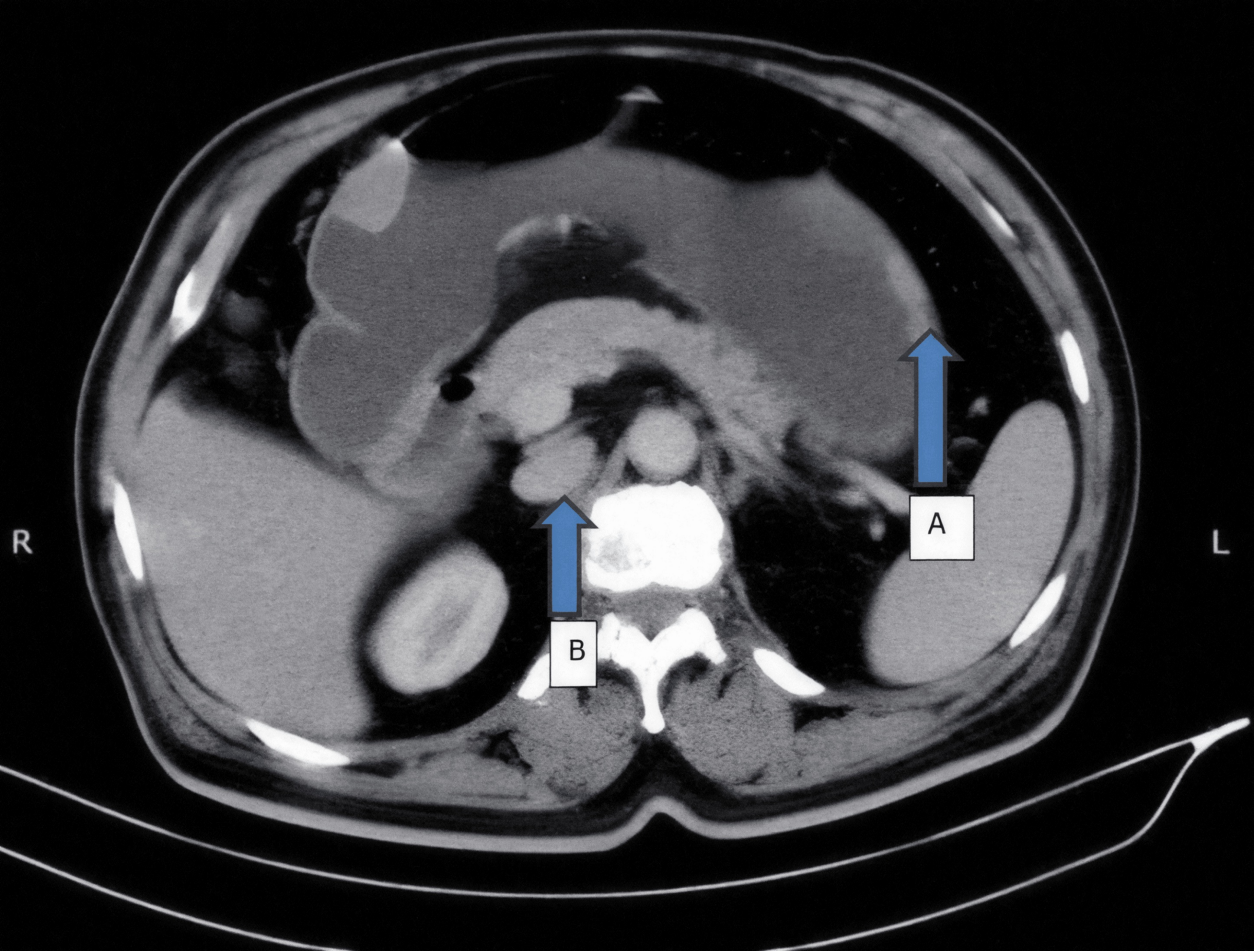
Contrast-enhanced CT of the abdomen revealing (A) heterogeneous enhanced polypoid lesions within the gastric antrum and fundus, and (B) a soft tissue mass lesion of 1.7 x 1.6 cm visualized in the retroperitoneal area.

Contrast-enhanced computed tomography (CECT) of the abdomen revealed a heterogeneous enhanced polypoid lesion within the gastric antrum and fundus with a soft tissue mass lesion measuring 1.7 x 1.6 cm in the retroperitoneal area (Figure 3). In view of the patient’s history of lower abdominal trauma several weeks prior to presentation, the lesion was initially interpreted as a possible infected hematoma. Multiple enlarged bilateral inguinal and external iliac lymph nodes were also noted.

**Figure 4:**
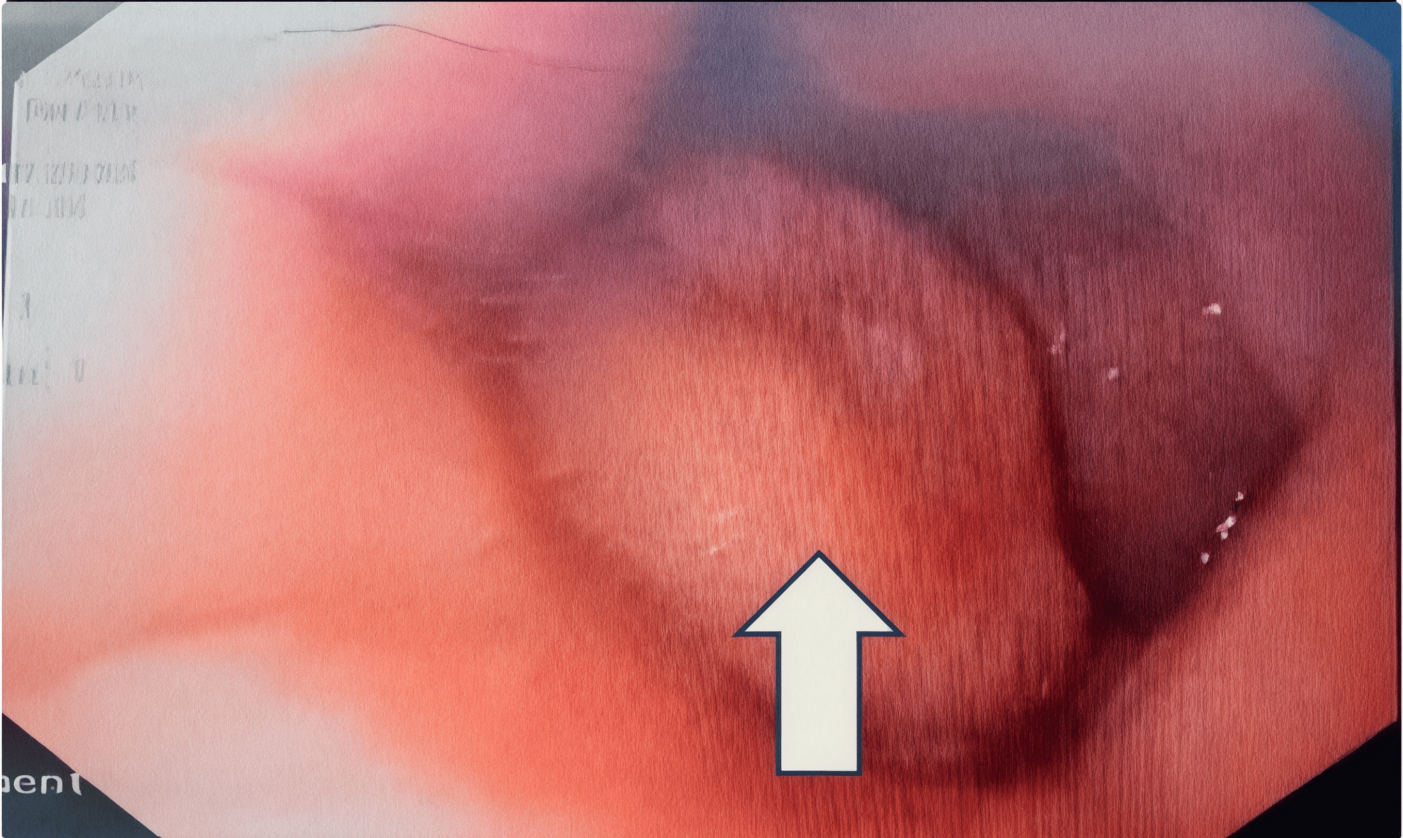
Upper endoscopy conducted to evaluate for gastric metastases showed a polypoidal lesion.

Additional findings on CECT included smooth polypoid lesions within the gastric antrum and fundus. A soft-tissue density measuring approximately 1.7 × 1.6 cm was also identified in the retroperitoneal region. Upper gastrointestinal endoscopy was subsequently performed to further evaluate the gastric metastases, which revealed a polypoidal lesion, as shown in Figure 4.

To establish a definitive diagnosis, biopsies were obtained from the scrotal wall lesion, gastric polyps, and one of the cutaneous nodules.

**Figure 5:**
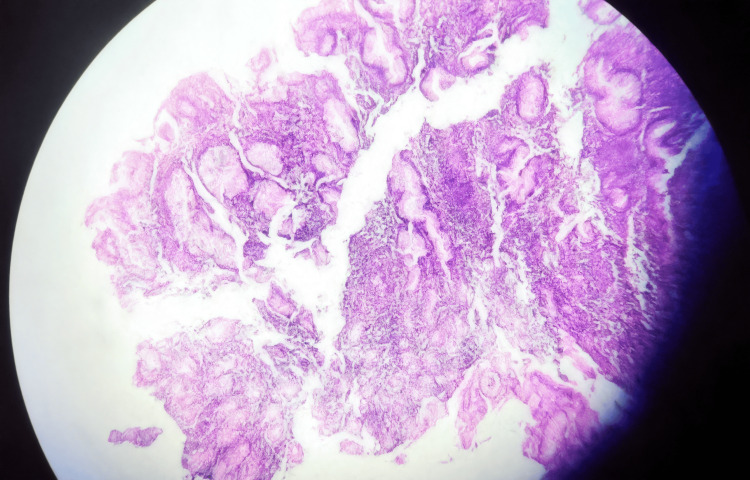
Low-magnification microscopy revealed medium-to-large atypical cells arranged in sheets.

**Figure 6:**
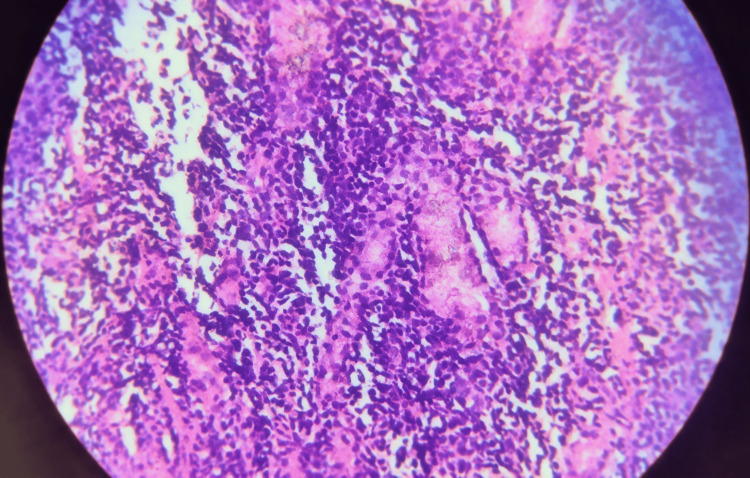
High-magnification microscopy showed cells with irregular nuclear contours, prominent nucleoli, and moderate cytoplasm.

Histopathological examination from all three sites demonstrated diffuse infiltration by medium-to-large atypical cells arranged in sheets on light microscopy. These cells exhibited irregular nuclear contours, prominent nucleoli, and moderate cytoplasm under high magnification. Figures 5, 6 show the low and high-magnification microscopy findings, respectively.

Immunohistochemical analysis revealed strong positivity for myeloperoxidase and CD34, confirming myeloid lineage. The Ki-67 proliferation index was markedly elevated at greater than 80%. Leukocyte common antigen showed weak membranous positivity, while tumor cells were negative for cytokeratin, CD3, CD20, and CD14.

Based on these findings, a diagnosis of multifocal myeloid sarcoma was established.

The patient was subsequently referred to the hematology department for further evaluation. Bone marrow examination was planned to assess systemic leukemia.

Induction chemotherapy using cytarabine and daunorubicin (7+3 protocol) was initiated. Localized radiotherapy was also administered to the scrotal lesion.

During the course of treatment, the patient developed febrile neutropenia followed by progressive respiratory distress requiring intensive care management. Despite aggressive supportive therapy, the patient developed multiorgan dysfunction and died four weeks after initiation of chemotherapy.

## Discussion

Myeloid sarcoma represents a localized accumulation of immature myeloid cells forming a solid tumor outside the bone marrow. The condition may occur concurrently with AML, represent relapse of previously treated leukemia, or appear as an isolated lesion preceding systemic leukemia [6].

The mechanisms underlying extramedullary infiltration are not fully understood but are believed to involve altered expression of adhesion molecules and chemokine receptors that allow leukemic blasts to migrate from the bone marrow into peripheral tissues. Once localized within these tissues, the malignant cells proliferate and form tumor masses [7].

The most frequently reported sites of involvement include the skin, lymph nodes, bone, and soft tissues. Less commonly affected organs include the gastrointestinal tract, central nervous system, breast, and genitourinary system [6].

Within the genitourinary tract, testicular involvement is particularly rare. Systematic reviews of the literature indicate that most reported cases of testicular myeloid sarcoma occur as isolated case reports, emphasizing the uncommon nature of this presentation [2]. Similar reports have also described cases in pediatric populations and infants, demonstrating that the condition may occur across a wide age range [5].

Clinically, testicular or scrotal myeloid sarcoma may present with nonspecific symptoms such as scrotal swelling, pain, or mass lesions. These manifestations frequently mimic more common conditions such as infection, inflammatory disease, or primary testicular tumors, often leading to initial misdiagnosis [3,4].

In the present case, the patient underwent multiple incisions and drainage procedures for a presumed scrotal abscess before the underlying malignancy was recognized. The persistence of swelling despite treatment and the appearance of cutaneous nodules prompted further evaluation.

Radiological imaging is useful for determining the extent of disease but is generally nonspecific. Findings may include solid enhancing masses or diffuse infiltration of soft tissues. The presence of lesions at multiple anatomical sites should raise suspicion for systemic disease or extramedullary leukemia.

Definitive diagnosis relies on histopathological examination with immunohistochemical analysis. Tumor cells typically express markers associated with myeloid differentiation, such as myeloperoxidase, CD34, CD117, CD33, and CD68. These markers are essential for distinguishing myeloid sarcoma from other malignancies, including lymphoma and poorly differentiated carcinoma [6].

Management strategies generally follow treatment protocols used for AML. Systemic chemotherapy with cytarabine-based regimens remains the cornerstone of therapy. Radiotherapy may be utilized for local disease control or symptomatic relief, while hematopoietic stem cell transplantation may be considered in selected patients depending on disease stage and overall clinical condition [7].

Despite treatment, the prognosis of myeloid sarcoma remains guarded, particularly when diagnosis is delayed or when disease involvement is multifocal. Several studies report a median survival of less than one year in advanced cases [8].

This case highlights the aggressive clinical behavior of myeloid sarcoma and underscores the importance of maintaining a high index of suspicion when evaluating persistent or atypical scrotal swellings.

## Conclusions

Myeloid sarcoma presenting as persistent scrotal swelling is an exceptionally rare clinical entity that may closely resemble common inflammatory or infectious conditions. Misdiagnosis can lead to delays in appropriate treatment and may adversely affect patient outcomes.

Clinicians should consider the possibility of hematologic malignancies in patients with treatment-refractory scrotal masses or atypical clinical features. Early biopsy, histopathological evaluation, and multidisciplinary management are essential for establishing an accurate diagnosis and initiating appropriate therapy.
